# Supplementary material for: Coupled binding mechanism of three sodium ions and aspartate in the glutamate transporter homologue GltTk
Source: Nat Commun. 2016 Nov 10;7:13420. doi: 10.1038/ncomms13420 (PMC5110648; doi:10.1038/ncomms13420)
Supplement: Supplementary Information — Supplementary Figures 1-5, Supplementary Tables 1-2 and Supplementary Note 1. [file ncomms13420-s1.pdf]

Supplementary Information

Supplementary Figures

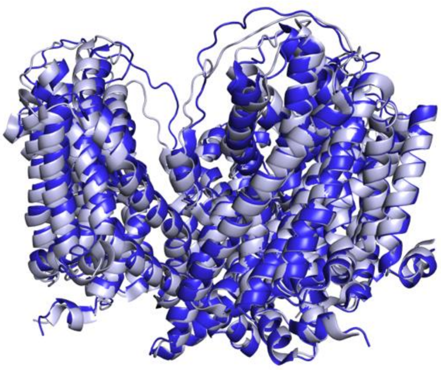

Supplementary Figure 1. Superposition of Glt_{Tk}^{sub} (blue) and Glt_{Tk}^{apo} (light blue). Both structures represent the outward-facing conformation.

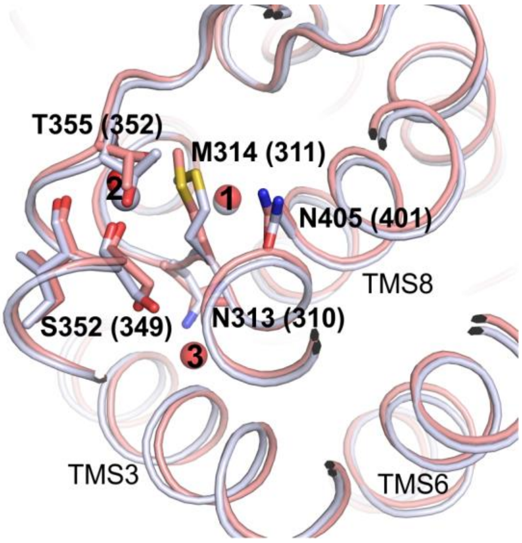

Supplementary Figure 2. Superposition of Glt_{Tk}^{sub} (red) with Tl-bound Glt_{Ph} (gray) (pdb code: 2NWX). Binding sites for sodium and thallium numbered 1 to 3. Sites 1 and 2 nearly identical, position 3 is observed only with sodium (this study)

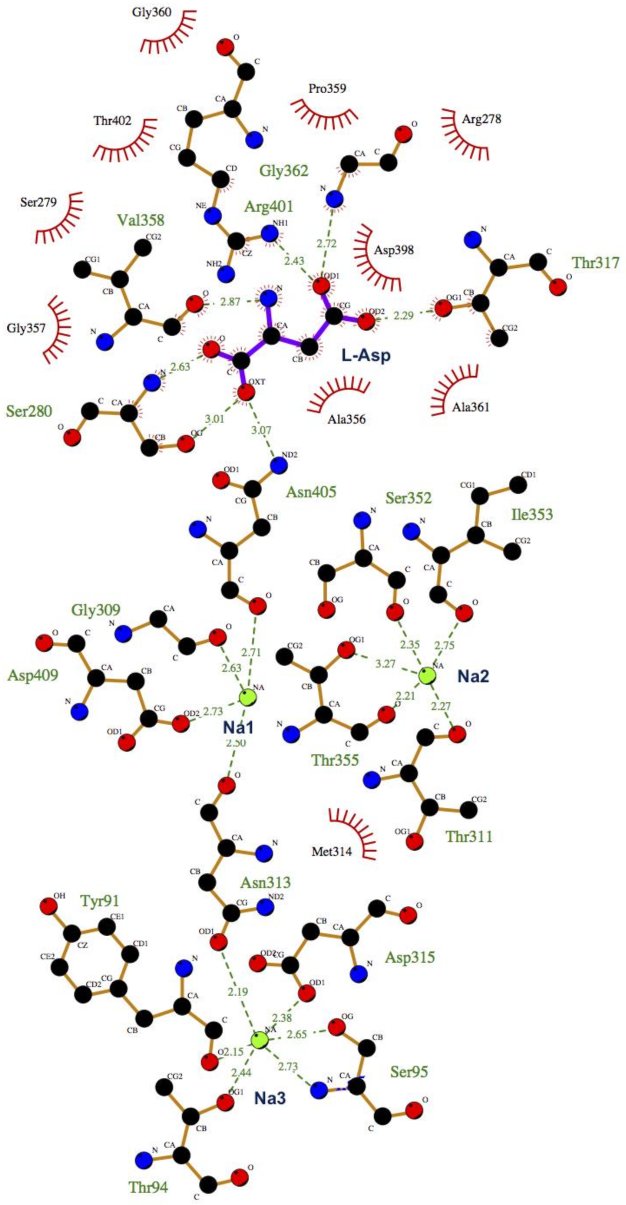

Supplementary Figure 3. Schematic representation of protein interactions with the ligands bound to Glt_{Tk}. L-Asp in purple, sodium ions in green. All distances in Å. Note the coupling between Na^+3, N313, Na^+1, N405 and L-Asp. Image was generated with Ligplot+[31].

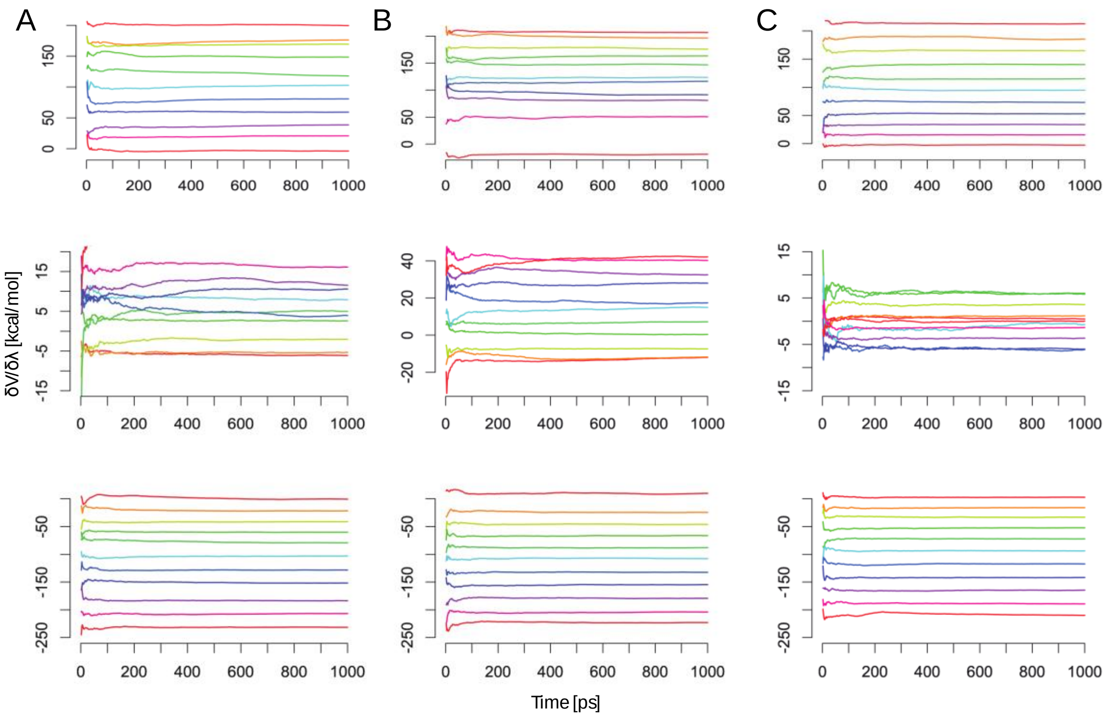

Supplementary Figure 4. Convergence analysis of the forward MD/TI calculations along time. The values correspond to the time evolution of the cumulative δV/δλ averages. Interaction free energy of the Na2 (A) and Na3 (B) in the complex. (C) Interaction free energies for the $Na^+ \rightarrow H_2O$ transformation in the bulk. Panels from top to bottom: discharging, van der Waals and recharging transformations. Different colors stand for the eleven intermediate λ values.

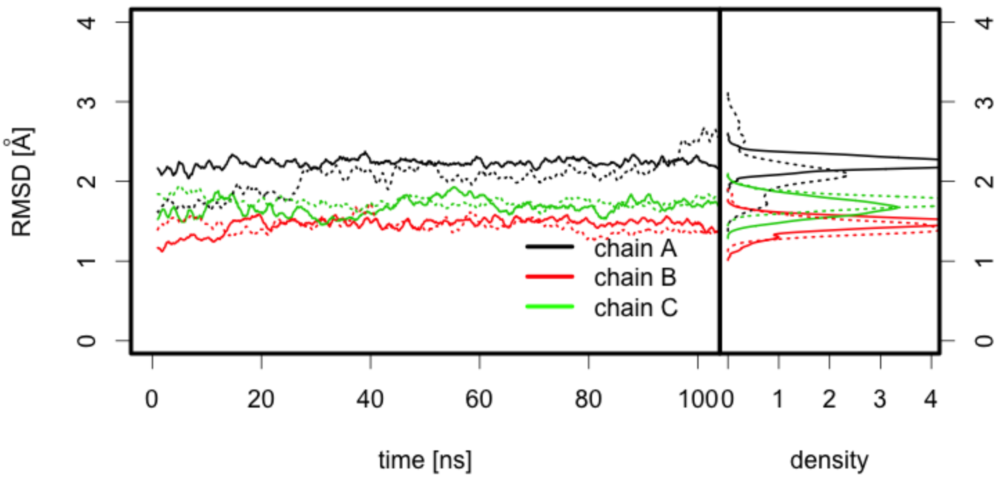

Supplementary Figure 5. RMSD of the protein backbone (Cα, N, O) during 100 ns of simulation for each monomer in the presence (solid) and absence (dashed) of ligands. The systems showed no large conformational changes after 20 ns, which indicates that the models were stable on these time scales.

Supplementary tables

		monomer A		monomer B		monomer C	
		crystal	MD	crystal	MD	crystal	MD
Na1	ASN 313 (O)	2.4	2.4 ± 0.1	2.4	2.4 ± 0.1	2.6	2.5 ± 0.2
	GLY 309 (O)	2.5	2.4 ± 0.1	2.5	2.4 ± 0.2	2.6	2.5 ± 0.2
	ASN 405 (O)	2.5	2.4 ± 0.2	2.6	2.4 ± 0.2	2.5	7.2 ± 0.3
	ASP 409 (O_2)	2.7	2.3 ± 0.1	2.6	2.3 ± 0.1	2.6	2.4 ± 0.1
	ASP 409 (O_1)	2.8	2.3 ± 0.1	2.7	2.3 ± 0.1	2.7	2.3 ± 0.1
Na2	SER 352 (O)	2.2	3.2 ± 0.4	2.4	(1)	2.2	4.6 ± 0.4
	THR 355 (O)	2.2	2.4 ± 0.2	2.3	(1)	2.1	2.5 ± 0.3
	THR 311 (O)	2.3	2.4 ± 0.2	2.3	(1)	2.4	4.5 ± 0.9
	MET 314 (S)	2.8	5.2 ± 0.4	3.1	(1)	2.9	4.5 ± 0.6
	ILE 353 (O)	2.5	2.4 ± 0.2	2.6	(1)	2.6	4.3 ± 0.7
Na3	ASP 315 (O_1)	2.0	2.3 ± 0.3	2.3	2.3 ± 0.2	2.3	2.4 ± 0.3
	SER 95 (N)	2.2	3.0 ± 0.2	2.4	3.0 ± 0.2	2.5	3.0 ± 0.2
	TYR 91 (O)	2.1	2.4 ± 0.2	2.2	2.4 ± 0.1	2.2	2.4 ± 0.1
	THR 94 (OG1)	2.2	2.4 ± 0.1	2.4	2.4 ± 0.1	2.3	2.4 ± 0.1
	SER 95 (OG)	2.6	2.8 ± 0.4	2.5	2.8 ± 0.3	2.4	2.8 ± 0.4
	ASN 313 (O_1)	2.7	2.3 ± 0.1	2.4	2.3 ± 0.1	2.3	2.3 ± 0.1

Supplementary Table 1 List of protein residues coordinating the three Na^+ ions in each monomer. Average Na^+-O distances obtained from 100 ns MD simulation. Values from MD simulation were compared with the distances in the crystallographic model. (1) No data was available for Na^+ ion in Na2 in monomer B since this left spontaneously the binding site.

	$-\Delta\Delta G_{int}$ (fwd)	$\Delta\Delta G_{int}$ (bwd)	ΔG_{tr}	ΔG_b
**Na2 (Na1,Na3,Asp)**	1.3 ± 3.6	-4.3 ± 3.6	5.0	3.7 ± 5.1
**Na3**	-26.8 ± 3.6	-19.8 ± 3.6	5.3	-18.0 ± 5.1
**Na1**	-16.1 ± 3.7	-15.5 ± 3.7	5.1	-10.7 ± 5.2
**Na1 (Na3)**	-7.6 ± 3.6	-8.1 ± 3.7	5.0	-2.9 ± 5.2

Supplementary Table 2. Binding free energies (**ΔG_b** in kcal/mol) for the Na^+ ions in Na1, Na2, and Na3 sites in the presence/absence of other ligands. The presence of other ligands in the binding site is shown in parentheses. ΔG_b is obtained as the sum of the translocation free energy of the ion from the bulk to the binding site, $\Delta\Delta G_{int}$ (averaged between the forward (fwd) and backward (bwd) transformation), and the change in translational free energy upon binding, ΔG_{tr}. Negative values correspond to a spontaneous $H_2O \rightarrow Na^+$ transformation. The differences in the forward and reverse calculations can be due to minor rearrangements in the binding sites during the transformation process. Errors represent standard errors, obtained from block averaging.

Supplementary note 1

Impact of the ligands in the dynamics around the HP2 region

The presence or absence of ligands in the binding site of the Glt_{Tk} showed differences at the local level. In these simulations where no restraints were applied, the average RMS values associated to the protein backbone were on the order of 2.0-3.1 Å and in general the presence/absence of the ligands has little effect at the global level (**Supplementary Fig. 5**). However, as anticipated from previous studies[15,22] , the absence of ligands in the binding site enhanced the fluctuations around the HP2 region as shown by the analysis of the protein residue fluctuations (**Fig. 5**). The relationship between the dynamics of the binding site and the HP2 hairpin was further supported by the rapid destabilization of the Na^+2 ion in the monomer B during the equilibration process.

In contrast to the less stable Na2 site, Na^+1 and Na^+3 are tightly bound within their binding sites. Five atoms coordinate the Na^+ ion in Na1, while 6 atoms coordinate the ion in Na3 site. The ion-ligand distances for the Na1 and Na3 sites observed in this and other available crystal structures of aspartate transporter were maintained along the whole trajectory (**Supplementary Table 1**). Even in the absence of the Na^+ ion in Na2 site in the monomer B, the Na^+ coordination number and stability in Na1 and Na3 sites were almost identical to the other two monomers (**Supplementary Table 1)**, which indicates that the opening of the HP2 gate has a small impact on the Na1 and Na3 sites.

Binding free energies

Free energy differences were calculated for Na1, Na2, and Na3 binding sites. Relative Na^+ binding free energies revealed that the Na3 site is the most stable site. As shown in **Supplementary Table 2**, the free energy difference associated to the loss of translational entropy was similar for all Na sites being around 5.0-5.3 kcal/mol. More importantly, the binding free energy of the Na3 site is -18 kcal/mol, implicating a strong binding site, whereas binding to the Na2 site is only weak at most (the affinity for water is actually larger, at least in the case we studied where all other sites are occupied). The explanation to this difference can be attributed to the higher coordination number for the Na^+ ion in the Na3 site (the Na^+ ion in Na2 site has one coordination atom less compared to the Na3 site) and also to the higher fluctuations of residues surrounding the Na2 site that are part of the flexible HP2 hairpin. The Na1 site is also more stable than the Na2 site, but less stable compared to the Na3 site by about 7 kcal/mol. Note that the stability of the Na1 site is reduced when the Na3 site is already occupied, likely due to electrostatic repulsion between the bound ions. Although free energy calculations are very sensitive to the structure, simulation conditions and force field parameters, they provide useful information about the relative preference among the Na sites. Our results, which are in the same line as previous

free energy calculations[15,22], support the binding mechanism in which the Na2 site is the last to be filled during the extracellular half-cycle of glutamate/aspartate co-transport.
